# Supplementary material for: Nursing students admitted through the affirmative action system display similar performance in professional and academic trajectories to those from the regular path in a public school in Brazil
Source: PLoS One. 2022 Mar 2;17(3):e0264506. doi: 10.1371/journal.pone.0264506 (PMC8890649; doi:10.1371/journal.pone.0264506)
Supplement: Attachment — Submitted filename: Response to reviewers .docx [file pone.0264506.s002.docx]

February 10, 2022

Emily Chenette

Editor-in-Chief

*PLOS ONE*

Dear Editor:

I would like to resubmit the attached manuscript titled “**Nursing students admitted through the affirmative action system display similar performance in professional and academic trajectories to those from the regular path in a public school in Brazil**.” The manuscript ID is PONE-D-21-11217.

The manuscript has been carefully rechecked, and the appropriate changes have been made in accordance with the referees’ suggestions. The changes have been highlighted in yellow for the convenience of the reviewers. Responses to the reviewers’ comments have been prepared and are included below.

We thank the referees for their thoughtful suggestions and insights, which have enriched the manuscript and produced a more balanced and better account of the research. We hope that the revised manuscript is now suitable for publication in *PLOS ONE*.

We look forward to your reply.

Sincerely,

Marize Lima de Sousa Holanda Biazotto

Graduation Program in Health Sciences, University of Brasília (UnB), Brasília, Federal District, Brazils

Faculdade de Medicina Faculdade de Ciências de Saúde Campos Univ. Darcy Ribeiro s/n - Asa Norte, Brasília - DF, 70910-900

Phone: +55 61999639496

Email: marizebiazo@hotmail.com

**Response to reviewers**

**Reviewer #1:**

There was an interesting revision of the paper. However, Authors introduced now the relevant element of Propensity Scores matching with the expected details. I urge Authors to fix this minor issue.

**Response:** Thank you for the comment and careful appraisal of the study. We clarify the description of the propensity score-matching in Methods as quoted below.

“To analyze the dependent variables in the survey questions, a propensity score matching for social quota system admission was performed applying a logit regression model adjusted for sex and time after nursing school completion was used. The EZR software version 1.54 (Saitama Medical Center, Jichi Medical University, Japan) with a 1:1 pair-matching ratio without replacement on the logit of the propensity score was employed in this analysis using a caliper of 0.2 width.”

**Reviewer #2:**

Thanks for revising the paper extensively. The changes made the paper more insightful and more convincing. The only issue that I'd like to raise is related to Table 1 (page 12).

I think the errors in the table are still uncorrected. The row on nursing school completion above four years says that 14 regular students and 21 quota students took longer than four years to complete school. However, if 150 regular students and 103 quota students graduated, then the percentage taking more than four years should be 9.3% for regular students and 20.4% for quota students.

The errors concern either the percentages OR the numbers in the table note. If the percentages, then also update/correct the statement on page 11 that "there was no significant difference regarding ... the time taken to complete nursing school (p = 0.837)."

**Response:** Thank you for the comment and careful appraisal of the study. The absolute values of regular and quota students taking more than four years to complete the nursing school were inverted in table 1, and we corrected this issue. The percentage of students taking more than four years to complete the nursing school was 14.0% for regular students (21/150) and 13.6% for the quota students (14/103).

We also changed the statement on page 11, as quoted below.

“There was no significant difference regarding nursing school attrition (p = 0.629), the time taken to complete nursing school (p = 0.837), and students taking more than four years to complete the nursing school (p = 0.926).”
